# Simultaneous targeting of EGFR, HER2, and HER4 by afatinib overcomes intrinsic and acquired cetuximab resistance in head and neck squamous cell carcinoma cell lines

**DOI:** 10.1002/1878-0261.12197

**Published:** 2018-05-01

**Authors:** Ines De Pauw, Filip Lardon, Jolien Van den Bossche, Hasan Baysal, Erik Fransen, Vanessa Deschoolmeester, Patrick Pauwels, Marc Peeters, Jan Baptist Vermorken, An Wouters

**Affiliations:** ^1^ Center for Oncological Research (CORE) University of Antwerp Wilrijk Belgium; ^2^ StatUa Center for Statistics University of Antwerp Belgium; ^3^ Department of Pathology Antwerp University Hospital Edegem Belgium; ^4^ Department of Medical Oncology Antwerp University Hospital Edegem Belgium

**Keywords:** afatinib, cetuximab, epidermal growth factor receptor, head and neck squamous cell carcinoma, human papillomavirus, resistance

## Abstract

The epidermal growth factor receptor (EGFR, HER1) is a therapeutic target in head and neck squamous cell carcinoma (HNSCC). After initial promising results with EGFR‐targeted therapies such as cetuximab, therapeutic resistance has become a major clinical problem, and new treatment options are therefore necessary. Moreover, the relationship between HER receptors, anti‐EGFR therapies, and the human papillomavirus (HPV) status in HNSCC is not fully understood. In contrast to first‐generation EGFR inhibitors, afatinib irreversibly inhibits multiple HER receptors simultaneously. Therefore, treatment with afatinib might result in a more pronounced therapeutic benefit, even in patients experiencing cetuximab resistance. In this study, the cytotoxic effect of afatinib as single agent and in combination with cisplatin was investigated in cetuximab‐sensitive, intrinsically cetuximab‐resistant, and acquired cetuximab‐resistant HNSCC cell lines with different HPV status under normoxia and hypoxia. Furthermore, the influence of cetuximab resistance, HPV, and hypoxia on the expression of HER receptors was investigated. Our results demonstrated that afatinib was able to establish cytotoxicity in cetuximab‐sensitive, intrinsically cetuximab‐resistant, and acquired cetuximab‐resistant HNSCC cell lines, independent of the HPV status. However, cross‐resistance between cetuximab and afatinib might be possible. Treatment with afatinib caused a G_0_/G_1_ cell cycle arrest as well as induction of apoptotic cell death. Additive to antagonistic interactions between afatinib and cisplatin could be observed. Neither cetuximab resistance nor HPV status significantly influenced the expression of HER receptors in HNSCC cell lines. In contrast, the expression of EGFR, HER2, and HER3 was significantly altered under hypoxia. Oxygen deficiency is a common characteristic of HNSCC tumors, and these hypoxic tumor regions often contain cells that are more resistant to treatment. However, we observed that afatinib maintained its cytotoxic effect under hypoxia. In conclusion, our preclinical data support the hypothesis that afatinib might be a promising therapeutic strategy to treat patients with HNSCC experiencing intrinsic or acquired cetuximab resistance.

**Abbreviations**
AnnV: annexin V‐FITC
CI: combination index
EGFR: epidermal growth factor receptor
HIF: hypoxia‐inducible factor
HNSCC: head and neck squamous cell carcinoma
HPV: human papillomavirus
mAb: monoclonal antibody
MFI: mean fluorescence intensity
PI: propidium iodide
R/M: recurrent/metastatic
SRB: sulforhodamine B
TKI: tyrosine kinase inhibitor

## 1 Introduction

Targeted therapies are at the forefront of personalized medicine in cancer treatment. Thanks to our rapidly expanding understanding of the molecular biology of cancer, an increasing number of patients are currently considered as candidates for treatment with molecular targeted pharmaceuticals. Interestingly, the epidermal growth factor receptor (EGFR, HER1) plays an integral role in the tumorigenesis and is richly expressed in a wide range of malignancies, including head and neck squamous cell carcinoma (HNSCC). Increased or sustained activation of the EGFR signaling pathway can convert a normal cell to a malignant cell by providing sustained signals for cell proliferation, anti‐apoptotic signaling, angiogenesis, and metastasis), thus making it a compelling drug target (Mahipal *et al*., 2014).

Head and neck squamous cell carcinoma is the sixth most common cancer in the world, and despite innovations in surgery and radiotherapy, overall 5‐year survival rates remain poor. HNSCCs are known to express high levels of EGFR, which is associated with poor prognosis (Mahipal *et al*., 2014). Therefore, inhibition of aberrant activation of the EGFR signal transduction pathway has been a focus of research over the last decades and has led to the development of tyrosine kinase inhibitors (TKIs), including erlotinib and gefitinib, as well as monoclonal antibodies (mAbs) that prevent ligand binding and/or receptor dimerization, such as cetuximab and panitumumab (Zhang *et al*., 2007). Clinical trials in HNSCC of cetuximab, gefitinib, or erlotinib, as monotherapies, have yielded response rates of only 5–15% (Cohen *et al*., 2009).

Most cancer treatments are combinations of chemotherapeutic agents and/or radiotherapy. Therefore, it was expected that EGFR‐targeted agents would achieve their greatest efficacy in combination with traditional cytotoxic agents or irradiation. Indeed, cetuximab may enhance the effect of radiotherapy and platinum‐based drugs by (a) inhibiting cell proliferation, cell repopulation, DNA repair, and tumor oxygenation; (b) modulating cell cycle perturbation and cell accumulation in radiosensitive phases; and (c) inducing apoptosis and necrosis (Fan *et al*., 1993; Skvortsova *et al*., 2010). In addition, it has already been observed that irradiation treatment results in an increase in EGFR expression on cancer cells (Liang *et al*., 2003). Furthermore, cetuximab has additional immune‐based mechanisms of activity as it is able to stimulate antibody‐dependent cellular cytotoxicity and enhance cytotoxic T‐lymphocyte cross‐priming by dendritic cells (Kimura *et al*., 2007; Yang *et al*., 2013). Both chemotherapy and radiation can also initiate effective antitumor immunity.

As a result, cetuximab has been approved for the treatment of HNSCC in combination with either radiotherapy in the locoregionally advanced disease setting (leading to an absolute survival gain of 10% at 5 years) or with platinum‐based drugs plus 5‐fluorouracil in the recurrent/metastatic (R/M) disease setting (leading to a median survival of 10.1 months versus 7.4 months with chemotherapy alone; Bonner *et al*., 2006; Vermorken *et al*., 2008). Although the addition of targeted therapy improves overall survival, lack of durable efficacy due to drug resistance is a major clinical problem (Cohen, 2014). To date, no definitive biomarkers have been identified to predict the efficacy of EGFR‐targeted therapies in patients with HNSCC (Boeckx *et al*., 2013; Kim *et al*., 2017).

Therapeutic resistance may arise from mechanisms that can compensate for reduced EGFR signaling and/or mechanisms that can modulate EGFR‐dependent signaling. In literature, activation of HER2 signaling has been associated with cetuximab resistance as its signaling occurs through many of the same downstream effectors of EGFR (Quesnelle and Grandis, 2011; Yonesaka *et al*., 2011). Therefore, it has been suggested that inhibition of both EGFR and HER2 could be an effective strategy to overcome cetuximab resistance. In HNSCC, increased HER expression has been linked to poor outcomes, including decreased overall survival, locoregional relapse, and treatment failure (Ang *et al*., 2002; Ganly *et al*., 2007; Takikita *et al*., 2011). Furthermore, Wheeler *et al*. (2008) demonstrated that cetuximab‐resistant HNSCC cells manifested strong activation of HER2 and HER3. In addition, cetuximab resistance could be the result of constitutive activation of the EGFR pathway caused by shedding of the HB‐EGF ligand after activation of ADAM by a stimulus, possibly interleukin 8 (Boeckx *et al*., 2015a). This HB‐EGF ligand binds not only to EGFR but also to HER4. As a result, inhibitors that bind multiple HER receptors might counteract cetuximab resistance.

Despite the reported intrinsic and acquired resistance to EGFR‐targeting agents, interest in targeting EGFR for the treatment of HNSCC remains high, with new strategies, such as inhibitor combinations and novel irreversible or multitargeting inhibitors, currently being evaluated. The particular mode of activation of the HER network, involving ligand‐induced homo‐ and heterodimerization of the four HER receptors, has prompted a new approach to inhibit this complex network and prevent premature emergence of resistance (Boeckx *et al*., 2013; Shepard *et al*., 2008). The simultaneous inhibition of both partners in a HER dimer, using covalent binders that confer irreversible inhibition, represents one of these new paradigms. In contrast to the first‐generation EGFR inhibitors, afatinib is an irreversible HER family blocker that inhibits the enzymatic activity of EGFR, HER2, and HER4 (De Pauw *et al*., 2016; Li *et al*., 2008a; Minkovsky and Berezov, 2008; Solca *et al*., 2012). As HER3 is kinase‐inactive and requires obligate heterodimerization with other HER family receptors, afatinib also inhibits HER3‐mediated signal transduction. The increased inhibition scope of HER receptors by afatinib most likely leads to a more robust blockade of the HER network (Ioannou *et al*., 2013). Previous preclinical research demonstrated effective cytotoxic activity of afatinib in HNSCC cell lines and xenograft models (Young *et al*., 2015). Consequently, treatment with afatinib might result in a distinct and more pronounced therapeutic benefit.

Besides crosstalk among the different HER receptor tyrosine kinases, therapeutic resistance may also arise after prolonged exposure of cells to reduced oxygen levels (hypoxia) (Wouters *et al*., 2007). Hypoxia‐inducible factors (HIFs) are, for instance, able to activate the EGFR signaling pathway (Wouters *et al*., 2013). As HNSCC is often characterized by hypoxic regions and as there is a link between hypoxia and EGFR signaling, we consider it highly important to investigate the cytotoxic effect of afatinib under both normal and reduced oxygen conditions.

Recent observations show that the human papillomavirus (HPV) is a diagnostic marker for a separate entity of HNSCC with enhanced overall and disease‐free survival, but its use as a predictive marker has not been proven yet (Marur *et al*., 2010). Although there is a great geographical variation in incidence of HPV‐associated tumors, 2013 global statistics demonstrated a 36% overall prevalence of HPV in HNSCC (Liu *et al*., 2013), thereby representing a substantial proportion of patients with HNSCC, which seems to further increase over time (Mehanna *et al*., 2013). HPV oncogenes have not been demonstrated to influence anti‐EGFR antibody response, and therefore, cetuximab treatment should be administered independently of HPV status (Nagel *et al*., 2013; Pogorzelski *et al*., 2014). Nevertheless, recent molecular phenotyping demonstrated EGFR‐independent signaling in HPV‐related HNSCC, suggesting that HPV‐positive patients with HNSCC could be less responsive to EGFR inhibitors (Machiels *et al*., 2015; Seiwert *et al*., 2015). Seiwert *et al*. (2015) demonstrated that the mutational landscape of HPV‐positive and HPV‐negative HNSCC differs significantly, which may explain the distinct clinical behavior and prognosis. As a result, further studies are needed to investigate the clinical implications of the observed mutations, with respect to sensitivity to targeted therapies, radiation, and chemotherapy. Research has also demonstrated that the expression of HER2 and HER3 is significantly elevated in HPV‐positive HNSCC in comparison with HPV‐negative HNSCC (Pollock *et al*., 2015). Furthermore, expression of these HER2 and HER3 receptors has previously been associated with resistance to EGFR inhibitors in HNSCC (Erjala *et al*., 2006). Consequently, agents targeting multiple HER receptors may have the potential to effectively treat both HPV‐positive and HPV‐negative tumors and may be able to overcome intrinsic and/or acquired cetuximab resistance in HNSCC (Pollock *et al*., 2015).

The present study aims to provide preclinical data concerning the efficacy of afatinib in monotherapy as well as in combination with cisplatin in HNSCC cell lines with different sensitivity to cetuximab. As the possible association between HPV status and efficacy of EGFR inhibition is still unclear, both HPV‐negative and HPV‐positive cell lines were included. Furthermore, variations in expression of HER family members between cell lines according to cetuximab resistance status, oxygen condition, and HPV status were investigated. In addition, the molecular mechanisms underlying the cytotoxic effect of afatinib were assessed.

## 2 Materials and methods

### 2.1 Cell culture

Eleven human HNSCC cancer cell lines with different cetuximab sensitivity and HPV status were included in this study. Cal‐27 and UM‐SCC‐104 were obtained from American Type Culture Collection (ATCC, Rockville, MD, USA) and Merck Millipore (SA/NV, Overijse, Belgium), respectively. SC263 and SQD9 were kindly provided by Sandra Nuyts (University Hospital Leuven, Leuven, Belgium), and LICR‐HN1 and SCC22b were kindly provided by Olivier De Wever (Laboratory of Experimental Cancer Research, Ghent University Hospital, Ghent, Belgium). 93‐VU147‐T was provided by Josephine Dorsman (VU University Medical Center, Amsterdam, the Netherlands). All cell lines were HPV negative, with the exception of 93‐VU147‐T and UM‐SCC‐104. All cell lines were cultured in Dulbecco's modified Eagle's medium, supplemented with 10% fetal bovine serum, 1% penicillin/streptomycin, and 1% l‐glutamine (Life Technologies, Merelbeke, Belgium). Cells were grown as monolayers and maintained in exponential growth in 5% CO_2_/95% air in a humidified incubator at 37 °C. All cell lines were confirmed free of mycoplasma infection through regular testing (MycoAlert Mycoplasma Detection Kit, Lonza, Verviers, Belgium).

### 2.2 Oxygen conditions

Hypoxia (1% O_2_) was achieved in a Bactron IV anaerobic chamber (Shel Lab, Cornelius, OR, USA), as described previously (Wouters *et al*., 2009). After overnight incubation to allow attachment of cells, hypoxic conditions were initiated immediately after addition of the drug.

### 2.3 Cytotoxicity assays

Cell survival was assessed using the colorimetric sulforhodamine B (SRB) assay, as previously described (Limame *et al*., 2012; Pauwels *et al*., 2003). This assay assesses the number of viable cells after treatment, as it is not possible to make a distinction with this assay between inhibition of proliferation (cytostatic effect) and cell death (cytotoxic effect). Optimal seeding densities for each cell line were determined to ensure exponential growth during the whole duration of the assay. Cells were counted automatically with a Scepter 2.0 device (Merck Millipore SA/NV). After overnight incubation, cells were treated with cetuximab (0–50 nm, 168 h), afatinib (0–10 μm, 72 h), or afatinib in combination with cisplatin (0–10 μm, 24 h). Two sequential combination schedules were tested:

1. Afatinib for 72 h immediately followed by cisplatin for 24 h;
2. Cisplatin for 24 h immediately followed by afatinib for 72 h.

The pharmaceuticals, that is, cetuximab (anti‐EGFR mAb, Merck, Darmstadt, Germany) and cisplatin (Selleck Chemicals, Houston, TX, USA), were diluted in sterile PBS. Afatinib (EGFR‐TKI, Selleck Chemicals) was diluted in DMSO (Merck Millipore SA/NV), and further dilutions were made in cell culture medium. IC_50_ values (i.e., drug concentration causing 50% growth inhibition) were calculated using winnonlin software (Pharsight, Mountain View, CA, USA). Possible synergism between afatinib and cisplatin was determined by calculation of the combination index (CI) using the additive model as described by others (Deben *et al*., 2016; Jonsson *et al*., 1998; Valeriote and Lin, 1975). CI < 0.8, CI = 1.0 ± 0.2, and CI > 1.2 indicated synergism, additivity, and antagonism, respectively.

### 2.4 Generation of resistant cell clones

Generation of resistant cell clones was performed as described previously (Boeckx *et al*., 2015a). Cetuximab‐resistant variants were derived from the original cetuximab‐sensitive parental SC263 and SCC22b cell lines by continuous exposure to cetuximab, starting with the IC_50_ concentration of cetuximab. In parallel, controlled parental cells were exposed to the vehicle control (suffix PBS). After 10 dose doublings, dose–response studies were determined for each resistant cell line (suffix R). To examine whether acquired resistance was a transient or permanent effect, dose–response curves of cetuximab were re‐assessed in the resistant cell lines after 6 weeks in culture without cetuximab.

### 2.5 Expression analysis of HER family members

The cellular membrane expression level of EGFR, HER2, HER3, and HER4 under both normoxia and hypoxia was assessed using flow cytometry. Cells were fixed in 4% formaldehyde (10 min) under normoxia or hypoxia after EGFR, HER2, HER3, and HER4 PE‐conjugated antibody incubation (10 μL/10^6^ cells, 25 μg antibody in 1 mL, R&D Systems, Minneapolis, MN, USA). These receptor‐specific antibodies recognize and bind to the extracellular domain of the receptor. Corresponding isotype controls (respectively, rat IgG2A, mouse IgG2B, mouse IgG1 and mouse IgG2A, 10 μL/10^6^ cells, 50 μg antibody in 2 mL; R&D Systems) were included for all samples and served as negative controls. Dead cells were excluded from the analysis by staining with Live/Dead Fixable Far‐Red Dead Cell Stain Kit (Thermo Fisher Scientific, Merelbeke, Belgium). All samples were measured on a FACScan flow cytometer (BD Biosciences, Erembodegem, Belgium). Flow cytometric data were analyzed using flowjo v10.1 (TreeStar Inc., Ashland, OR, USA). The percentage of EGFR‐, HER2‐, HER3‐ and HER4‐positive cells (overton) was determined in comparison with the corresponding isotype control. Furthermore, the signal for aspecific binding was subtracted from the mean fluorescence intensities (=ΔMFI). This parameter indicates the amount of cellular membrane expression of EGFR, HER2, HER3, and HER4 on individual cells.

### 2.6 Assays for apoptosis and cell cycle distribution

After overnight incubation, cells were treated for 72 h with afatinib. As the sensitivity to afatinib strongly varied between the cell lines, afatinib concentrations were based on the outcome of the monotherapy experiments and corresponded with the IC_20_, IC_40,_ and IC_60_ values specific for each cell line under normoxia and hypoxia. Cell cycle distribution was determined immediately after 72 h of treatment with afatinib under both normoxia and hypoxia, using a CycleTEST™ PLUS DNA reagent kit (BD Biosciences). Induction of apoptotic cell death was investigated flow cytometrically using the annexin V‐FITC (AnnV)/propidium iodide (PI) assay (BD Biosciences). Both assays were performed on a FACScan flow cytometer and analyzed with flowjo v10.1.

Alternatively, the induction of apoptotic cell death was examined by real‐time measurements of caspase‐3/7 activity using the IncuCyte ZOOM live‐cell analysis instrument (Essen BioScience, Ann Arbor, MI, USA). After overnight incubation, cells were treated with afatinib. The IncuCyte caspase‐3/7 green apoptosis reagent (Essen BioScience) was added at the start of treatment at a final concentration of 2.5 μm. This caspase‐3/7 apoptosis reagent is cleaved by activated caspase‐3/7, which results in the release of a DNA dye and green fluorescent staining of nuclear DNA. Images were taken every 2 h from the start of treatment. Kinetic activation of caspase‐3/7 was monitored morphologically using live‐cell imaging and quantified using the IncuCyte basic analyzer (Essen BioScience).

### 2.7 Statistical analysis

We performed all experiments at least three times independently, unless otherwise stated. In cytotoxicity experiments and IncuCyte Caspase‐3/7 Green Apoptosis Assays, each condition was tested in triplicate in each of the three experiments. Flow cytometry experiments were independently performed three times with one sample for each condition. Results are presented as mean ± standard deviation. The effects of various conditions and treatments were studied using linear regression or linear mixed models in case of nonindependent observations. All models were fitted using a stepwise backward strategy, starting from a model with all fixed effects and their interaction. If the interaction term was not significant, a model with only the main was fitted. If one of these terms was significant, effect sizes were estimated. If the treatment effect was significant, a *post hoc* analysis with Tukey's correction for multiple testing was performed.

Effects of oxygen and resistance status on the expression of HER family members and afatinib's cytotoxic effect were modeled using a linear mixed model with oxygen status, resistance status, and their interaction as fixed effects. A random intercept for cell line was added to account for the dependence between observations within the same cell line. Furthermore, a separate analysis was performed to test for differences in HER status between HPV‐positive and HPV‐negative HNSCC cell lines. Linear regression models were fitted to study the effect of treatment, oxygen condition, and their interaction on the percentage of G_0_/G_1_ cells as well as AnnV+/PI− and AnnV+/PI+ cells. Regarding the combination experiments, differences in IC_50_ values were tested for significance with the Mann–Whitney *U*‐test.

graphpad prism 7 (Graphpad Software, La Jolla, CA, USA) was used for data comparison and artwork. All statistical analyses were performed in r version 3.3.2 (The R Foundation for Statistical Computing, Vienna, Austria). *P*‐values below 0.050 were considered significant.

## 3 Results

### 3.1 Identification of HNSCC cell lines with intrinsic cetuximab resistance and generation of cell lines with acquired cetuximab resistance

**Figure 1:**
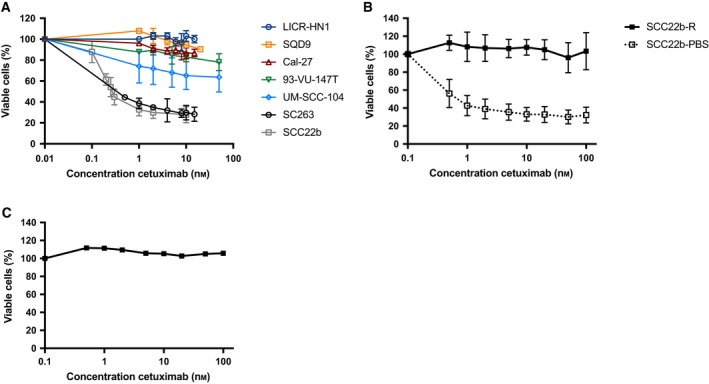
Dose–response curves of cetuximab (168 h) evaluated using the SRB assay. (A) Dose–response curves for HPV‐positive and HPV‐negative HNSCC cell lines. Five of seven HNSCC cell lines (i.e., LICR‐HN1, Cal‐27, SQD9, 93‐VU‐147T, and UM‐SCC104) were considered as intrinsically resistant to cetuximab versus SC263 and SCC22b, which were cetuximab sensitive. (B) Dose–response curves for isogenic cetuximab‐resistant (SCC22b‐R) and cetuximab‐sensitive (SCC22b‐PBS) HNSCC cell lines. (C) Dose–response curve for the cetuximab‐resistant cell line SCC22b‐R after 6 weeks of culture in drug‐free medium, followed by cetuximab treatment for 168 h. This graph represents one experiment executed in threefold.

We previously identified intrinsic resistance to cetuximab in several HNSCC cell lines such as LICR‐HN1 and Cal‐27 (Boeckx *et al*., 2014). A resistant daughter cell line was already generated from the cetuximab‐sensitive SC263 cell line, in order to establish acquired resistance to cetuximab (Boeckx *et al*., 2015a). Similarly, sensitivity to cetuximab therapy was investigated in an additional panel of HNSCC cell lines. The dose–response curves of the HNSCC cell lines for cetuximab are shown in Fig. 1. Based on these dose–response curves and the corresponding IC_50_ values, five of seven HNSCC cell lines (i.e., LICR‐HN1, Cal‐27, SQD9, 93‐VU‐147T, and UM‐SCC‐104) were considered as intrinsically resistant to cetuximab as the percentage of viable cells in these cell lines did not decrease below 50%. SC263 and SCC22b were identified as cetuximab sensitive (IC_50_ values of 0.12 ± 0.04 nm and 0.41 ± 0.23 nm, respectively).

As mentioned above, one acquired cetuximab‐resistant daughter HNSCC cell line was already generated during previous research (Boeckx *et al*., 2015a). An additional HNSCC cell line with acquired cetuximab resistance was developed from the human SCC22b HNSCC cell line, shown to be initially cetuximab sensitive. Resistant cells were characterized by performing cell proliferation assays upon exposure to cetuximab (Fig. 1B). A higher proliferation potential was observed in the acquired cetuximab‐resistant SCC22b‐R cells when treated with cetuximab compared to sensitive SCC22b‐PBS cells. Furthermore, the stability of cetuximab resistance was confirmed, as SC263‐R (Boeckx *et al*., 2015a) and SCC22b‐R (Fig. 1C) remained cetuximab resistant, even after culturing in drug‐free medium for 6 weeks.

### 3.2 Expression level of HER family members in a panel of HNSCC cell lines with different sensitivity to cetuximab

As afatinib inhibits multiple members of the HER receptor family, we examined the basal cellular membrane protein expression level of these HER family members under normoxic and hypoxic conditions in our panel of HNSCC cell lines with different sensitivity to cetuximab and HPV status.

**Figure 2:**
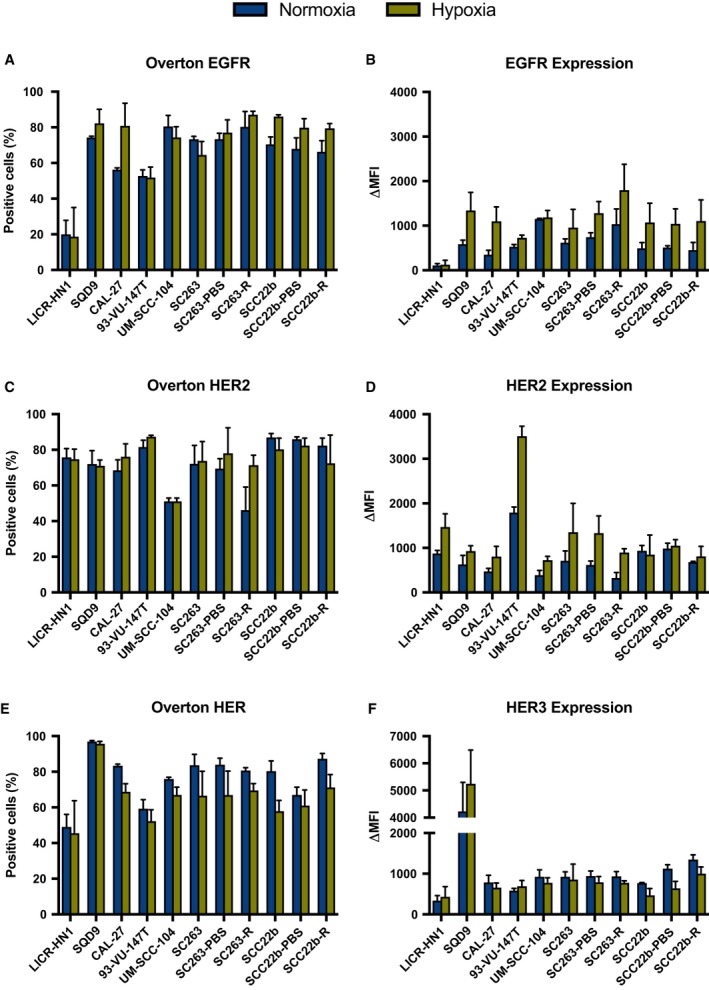
Protein levels of HER receptors under normoxic and hypoxic conditions in a panel of HNSCC cell lines with different sensitivity to cetuximab. The percentage of EGFR‐, HER2‐, and HER3‐positive cells (overton) are presented in A, C, and E, respectively. The expression levels of EGFR, HER2, and HER3 on the corresponding receptor‐positive cells (ΔMFI) are presented in B, D, and F, respectively. Protein levels were measured with the FACScan flow cytometer.

The majority of HNSCC cell lines showed high percentages of EGFR‐, HER2‐, and HER3‐positive cells (Fig. 2A,C,E). Furthermore, these receptor‐positive cells demonstrated high expression of EGFR, HER2, and HER3 (Fig. 2B,D,F). In contrast, HER4 expression was barely observed in any of the HNSCC cell lines tested, and when detected, HER4 expression levels were very low (data not shown). Remarkably, LICR‐HN1 demonstrated lower levels of EGFR expression in comparison with other HNSCC cell lines. Nevertheless, EGFR, HER2, and HER3 were highly expressed in all other HNSCC cell lines with ΔMFI above 346.7 ± 101.0, 326.3 ± 122.8, and 586.7 ± 54.1, respectively. Interestingly, the intrinsically cetuximab‐resistant cell lines 93‐VU‐147T and SQD9 demonstrated huge levels of, respectively, HER2 (ΔMFI = 1792.3 ± 125.6) and HER3 expression (ΔMFI = 4229.7 ± 1068.2) compared to the other HNSCC cell lines.

No significant differences in the percentages of EGFR‐, HER2‐, HER3‐, and HER4‐positive cells (*P* ≥ 0.143) and the ΔMFI of receptor‐positive cells (*P* ≥ 0.170) were observed between cetuximab‐sensitive, intrinsically cetuximab‐resistant, and acquired cetuximab‐resistant HNSCC cell lines. In addition, no significant effect of HPV status was detected for the percentage of EGFR‐, HER2‐, HER3‐, and HER4‐positive cells (*P* ≥ 0.302) as well as ΔMFI of receptor‐positive cells (*P* ≥ 0.110). Hence, the percentage EGFR‐, HER2‐, HER3‐, and HER4‐positive cells and ΔMFI of receptor‐positive cells seems to be cell line specific and independent of HPV status.

**Figure 3:**
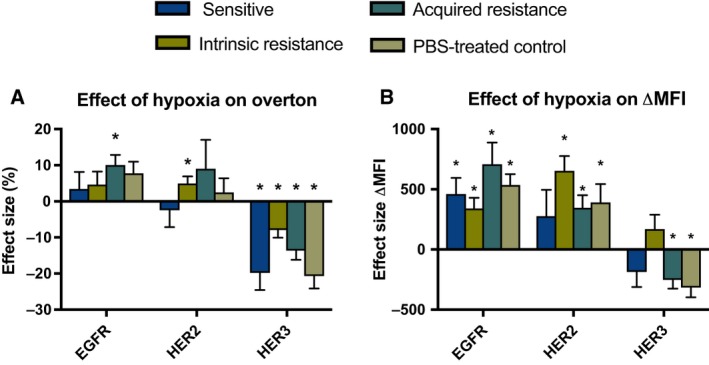
Effect sizes with standard errors for the influence of hypoxia on the expression of HER family members in HNSCC cell lines with different cetuximab resistance status. The effect size represents the difference in mean overton (A) and ΔMFI (B) between normoxia and hypoxia. Positive and negative effect sizes, respectively, indicate an increase and decrease under hypoxia compared to normoxia. The * indicates a significant *P*‐value for the main effect of oxygen condition.

Next, the effect of reduced oxygen levels on the expression of EGFR, HER2, HER3, and HER4 was evaluated. No significant interaction between oxygen condition and cetuximab resistance status on EGFR, HER2, HER3, and HER4 expression was found. When looking at the effect of oxygen availability, there was a significant increase in the percentage of EGFR‐positive cells (*P* = 0.006) and ΔMFI of EGFR‐positive cells (*P* < 0.001) under hypoxic conditions in all HNSCC cell lines (Table 1). The significant increase in overton for EGFR under hypoxia was mainly observed in acquired cetuximab‐resistant HNSCC cell lines (*P* = 0.016) (Fig. 3A). The significant increase in ΔMFI of EGFR‐positive cells under hypoxia was detected in cetuximab‐sensitive and intrinsically cetuximab‐resistant as well as acquired cetuximab‐resistant and PBS‐treated HNSCC cell lines (*P* ≤ 0.018) (Fig. 3B). Regarding HER2, the percentage of HER2‐positive cells increased under hypoxic conditions in all HNSCC cell lines (*P* = 0.065) (Table 1), especially in intrinsically cetuximab‐resistant HNSCC cell lines (*P* = 0.024) (Fig. 3A). Moreover, ΔMFI of HER2‐positive cells was significantly raised under hypoxic conditions in all HNSCC cell lines (*P* < 0.001). This significant increase under hypoxia was mainly noticed in intrinsically cetuximab‐resistant as well as acquired cetuximab‐resistant and PBS‐treated control HNSCC cell lines (*P* ≤ 0.050) (Fig. 3B). In contrast, for HER3, the percentage of positive cells was significantly decreased under hypoxia in all HNSCC cell lines (*P* < 0.001) (Table 1). Overall, no significant change in ΔMFI of HER3‐positive cells was observed under reduced oxygen levels (*P* = 0.425). However, taking the difference in cetuximab resistance status between cell lines into account, a significant decrease in ΔMFI under hypoxia was observed for HER3 in acquired cetuximab‐resistant and PBS‐treated control HNSCC cell lines (*P* ≤ 0.010) (Fig. 3B). Lastly, HER4 expression was not altered under hypoxic conditions (*P* ≥ 0.300) (data not shown).

**Table 1:** Effect sizes with standard errors for the influence of oxygen condition on the expression levels of EGFR, HER2, HER3, and HER4 in all HNSCC cell lines. The effect size is only reported if there was a significant difference in overton and ΔMFI of HER receptors between normoxia and hypoxia (*P*‐value ‘effect oxygen’). The effect size represents the difference in mean overton and ΔMFI between normoxia and hypoxia. Positive and negative effect sizes, respectively, indicate an increase and decrease under hypoxia compared to normoxia. The *P*‐value shows the significance, testing the null hypothesis that the effect size equals zero. *P*‐values ≤ ≤0.05 are indicated in bold. /, effect size was not shown in case there was no significant effect of oxygen condition on the outcome

	% Positive cells (overton)			Expression level (ΔMFI)		
	*P*‐value effect oxygen	Effect size (%)	*P*‐value effect size	*P*‐value effect oxygen	Effect size (ΔMFI)	*P*‐value effect size
EGFR	**0.006**	5.95 ± 2.03	**0.003**	**< 0.001**	463.35 ± 64.65	**< 0.001**
HER2	0.065	/	/	**< 0.001**	483.62 ± 80.93	**< 0.001**
HER3	**< 0.001**	−12.95 ± 1.95	**< 0.001**	0.425	/	/
HER4	0.377	/	/	0.300	/	/

Overall, these results demonstrated that HNSCC cell lines with different sensitivity to cetuximab contain a high percentage of EGFR‐, HER2‐, and HER3‐positive cells and that these receptors are generally highly expressed on these positive cells. Furthermore, hypoxia significantly affected the expression of these receptors. In contrast, cetuximab resistance and HPV status had no significant influence on the expression of HER receptors. This means that the HNSCC cell lines used in this study are a valid target candidate for treatment with afatinib, according to the target expression of EGFR and HER2.

### 3.3 Afatinib is able to overcome intrinsic and acquired cetuximab resistance in a panel of HNSCC cell lines under normal and reduced oxygen conditions

**Figure 4:**
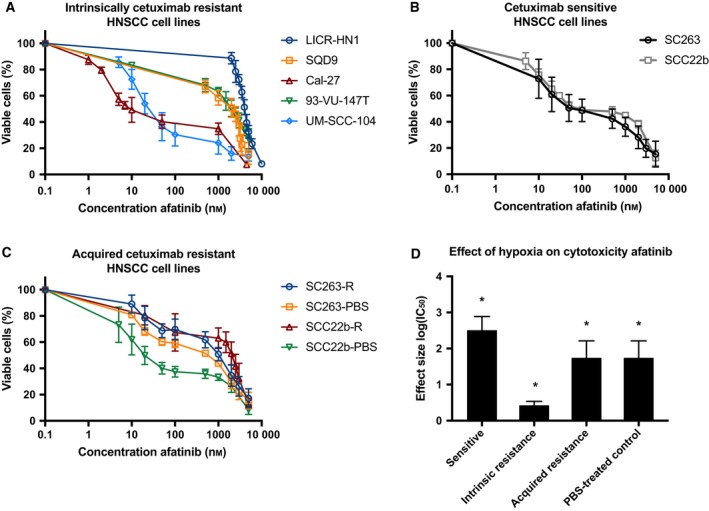
Afatinib's cytotoxicity in cetuximab‐sensitive and cetuximab‐resistant HNSCC cell lines. (A) Dose–response curves of HPV‐positive and HPV‐negative intrinsically cetuximab‐resistant HNSCC cell lines after exposure to afatinib for 72 h under normoxia. (B) Dose–response curves for the cetuximab‐sensitive HNSCC cell lines after exposure to afatinib for 72 h under normoxia. (C) Dose–response curves of acquired cetuximab‐resistant (suffix R) and corresponding cetuximab‐sensitive isogenic cell lines (suffix PBS) after exposure to afatinib for 72 h under normoxia. (D) Effect sizes with standard errors for the influence of hypoxia on afatinib's cytotoxic effect in HNSCC cell lines with different cetuximab resistance status. The average log(IC_50_) of afatinib was higher under normoxia compared to hypoxia in cetuximab‐sensitive, intrinsically cetuximab‐resistant, and acquired cetuximab‐resistant HNSCC cell lines. The effect size represents the difference in mean log(IC_50_) between normoxia and hypoxia. The * indicates a significant *P*‐value for the main effect of oxygen condition.

The cytotoxic effect of the irreversible HER family blocker afatinib was studied in cetuximab‐sensitive, intrinsically cetuximab‐resistant as well as acquired cetuximab‐resistant and PBS‐treated control HNSCC cell lines under both normoxia and hypoxia. A clear concentration‐dependent cytotoxic effect of afatinib (0–5000 nm) after 72 h of treatment was observed in all HNSCC cell lines (Fig. 4). The IC_50_ values for afatinib under normoxic conditions ranged from 19 ± 15 nm to 4040 ± 70 nm (Table 2). Neither cetuximab resistance (*P* = 0.750) nor the HPV status (*P* = 0.800) seems to influence the inhibitory potential of afatinib. However, certain intrinsically cetuximab‐resistant and acquired cetuximab‐resistant HNSCC cell lines demonstrated higher IC_50_ values compared to other intrinsically cetuximab‐resistant and acquired cetuximab‐resistant HNSCC cell lines used in this study. For instance, the intrinsically cetuximab‐resistant HNSCC cell lines Cal27 and UM‐SCC‐104 demonstrated considerably lower IC_50_ values compared to LICR‐HN1 and 93‐VU‐147T. Despite that statistical analysis did not reveal a significant influence of cetuximab resistance on the cytotoxicity of afatinib, these results indicate the possibility of cross‐resistance between cetuximab and afatinib.

**Table 2:** IC_50_ values and standard errors for HNSCC cell lines after incubation with afatinib for 72 h under normoxic and hypoxic conditions

Cell line	Cetuximab resistance status	HPV status	IC_50_ Afatinib 72 h (nm)	
			Normoxia (21% O_2_)	Hypoxia (1% O_2_)
LICR‐HN1	Intrinsically resistant	Negative	4040 ± 70	3750 ± 80
SQD9	Intrinsically resistant	Negative	1500 ± 310	1950 ± 310
Cal27	Intrinsically resistant	Negative	19 ± 15	7 ± 6
93‐VU147‐T	Intrinsically resistant	Positive	2210 ± 260	2350 ± 390
UM‐SCC‐104	Intrinsically resistant	Positive	34 ± 16	22 ± 7
SC263	Sensitive	Negative	89 ± 34	20 ± 11
SC263‐PBS	PBS‐treated control, sensitive	Negative	260 ± 110	28 ± 12
SC263‐R	Acquired resistant	Negative	680 ± 270	160 ± 60
SCC22b	Sensitive	Negative	170 ± 110	29 ± 18
SCC22b‐PBS	PBS‐treated control, sensitive	Negative	34 ± 19	22 ± 13
SCC22b‐R	Acquired resistant	Negative	2420 ± 370	225 ± 166

The cytotoxic effect of afatinib was maintained under reduced oxygen conditions (Table 2). Cetuximab resistance (*P* = 0.450) and HPV status (*P* = 0.630) were not associated with afatinib's cytotoxicity under hypoxic conditions. However, statistical analysis showed a significant interaction between resistance status and oxygen condition (*P* < 0.001). This implies that the difference in IC_50_ value for afatinib between the normoxia and hypoxia group varies across the different resistance statuses. As such, we analyzed the difference in IC_50_ value of afatinib between normoxia and hypoxia for cetuximab‐sensitive, intrinsically cetuximab‐resistant, and acquired cetuximab‐resistant HNSCC cell lines. The estimated effect sizes, showing the difference in mean log(IC_50_) between normoxia and hypoxia, are displayed in Fig. 4D. Significantly higher average log(IC_50_) values were observed under normoxia compared to log(IC_50_) values under hypoxia in cetuximab‐sensitive, intrinsically cetuximab‐resistant, and acquired cetuximab‐resistant HNSCC cell lines (*P* < 0.001). This means that afatinib demonstrated an increased cytotoxicity under hypoxic conditions. However, this effect of hypoxia on afatinib's cytotoxic activity was less pronounced in intrinsically cetuximab‐resistant cell lines.

Overall, afatinib showed a clear concentration‐dependent cytotoxic effect in cetuximab‐sensitive, intrinsically cetuximab‐resistant, and acquired cetuximab‐resistant HNSCC cell lines. Furthermore, our results demonstrate that therapeutic resistance to afatinib is not associated with HPV infection or prolonged exposure to hypoxia in our panel of HNSCC cell lines with different sensitivities to cetuximab.

### 3.4 Molecular mechanisms underlying the cytotoxic effect of afatinib

#### 3.4.1 Treatment with afatinib results in a G_0_/G_1_ cell cycle arrest

**Figure 5:**
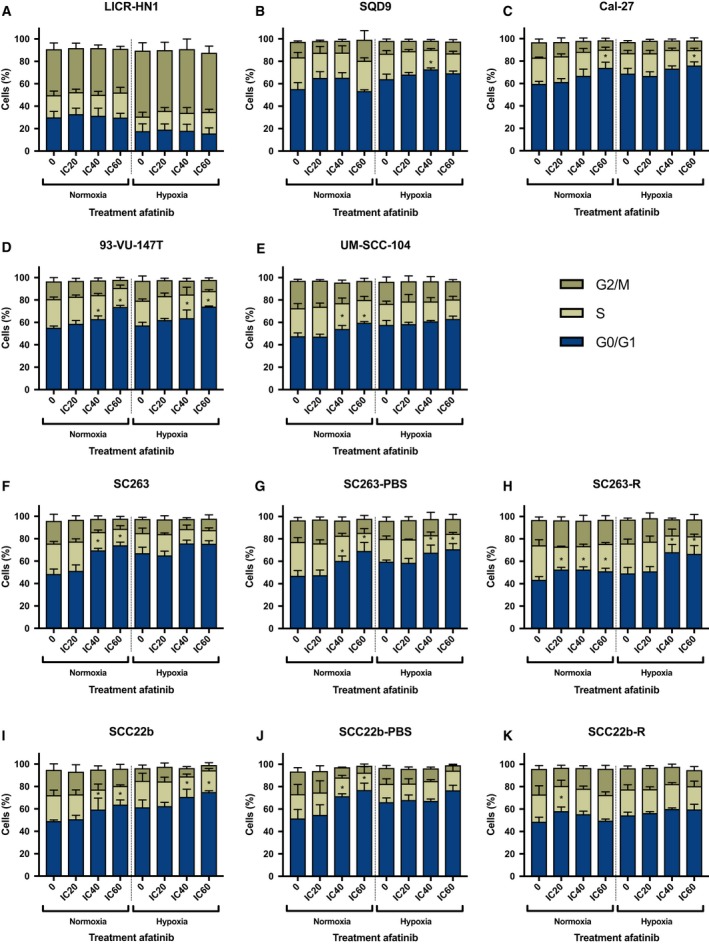
Cell cycle distribution in HNSCC cell lines after afatinib treatment (0 nm, IC_20_, IC_40_, IC_60_) under both normoxic and hypoxic conditions. Cells were stained with PI and DNA content was measured by flow cytometric analysis. Cells were divided into 3 groups: G_0_/G_1_ phase (2*n*), S phase (2*n*–4*n*), and G_2_/M phase (4*n*). Treatment with afatinib led to an increase of the proportion of cells in the G_0_/G_1_ phase of the cell cycle in the majority of HPV‐negative (A–C) and HPV‐positive (D, E) intrinsically cetuximab‐resistant HNSCC cell lines under both normoxia and hypoxia. This G_0_/G_1_ cell cycle arrest was also observed in cetuximab‐sensitive (F, I) as well as isogenic acquired cetuximab‐resistant and PBS‐treated control HNSCC cell lines (G, H, J, and K). *P‐value effect treatment on the percentage G_0_/G_1_ cells ≤ 0.050.

As presented in Fig. 5, treatment with afatinib under both normoxia and hypoxia led to an increase in the percentage of G_0_/G_1_ cells, accompanied by a decrease in the percentage of cells in the S and G_2_/M phases, in the majority of the HNSCC cell lines, irrespective of their sensitivity to cetuximab.

The effects of treatment and oxygen condition as well as their interaction on the percentage of G_0_/G_1_ cells were analyzed using linear regression analyses (Table 3). A significant interaction indicates that the effect of afatinib treatment on the percentage of G_0_/G_1_ cells is different under normoxia versus hypoxia. Moreover, it also implies that the effect of oxygen condition on the percentage of G_0_/G_1_ cells varies after treatment with different afatinib concentrations. The interaction between treatment and oxygen status was found to be significant in half of the HNSCC cell lines used in this study, indicating a cell line‐specific effect (Table 3). If the interaction was not significant, a model with the main effects of treatment and oxygen condition was performed. Thus, in absence of a significant interaction, the effect of treatment is valid under both normoxia and hypoxia. With regard to the effect of treatment under normoxia, afatinib induced a significant increase in the proportion of G_0_/G_1_ cells in all cell lines, except for LICR‐HN1. Compared to treatment under normoxia, the effect of afatinib on the G_0_/G_1_ cell cycle arrest was generally lower or equal under hypoxia in cetuximab‐sensitive and intrinsically cetuximab‐resistant HNSCC cell lines, except for SQD9. Remarkably, in the acquired cetuximab‐resistant cell lines, the increase in G_0_/G_1_ cells after treatment under normoxia was less pronounced compared to the isogenic PBS‐treated control cell lines, especially in the SCC22b‐R versus SCC22b‐PBS cell line. The influence of hypoxia on the G_0_/G_1_ cell cycle arrest induced by afatinib was variable in these acquired cetuximab‐resistant HNSCC cell lines. For instance, treatment with afatinib under hypoxia, compared to normoxia, resulted in a more pronounced G_0_/G_1_ cell cycle arrest in the acquired cetuximab‐resistant SC263‐R cell line but not in the SCC22b‐R cell line.

**Table 3:** *P*‐values for the interaction of treatment and oxygen condition on the percentage of cells in the G_0_/G_1_ phase of the cell cycle in HNSCC cell lines. If the interaction was not significant (*P* > 0.050), a model with the main effects of treatment and oxygen condition was fitted to test the significance of treatment effect and oxygen condition separately. NE: main effects were not estimated in case of a significant interaction between treatment and oxygen. Values ≤ ≤0.05 are indicated in bold

Cell line	*P*‐value interaction treatment and oxygen condition	*P*‐value main effect treatment	*P*‐value main effect oxygen condition
LICR‐HN1	0.991	0.722	**< 0.001**
SQD9	**0.029**	NE	NE
Cal27	0.433	**< 0.001**	**< 0.001**
93‐VU‐147T	0.776	**< 0.001**	0.149
UM‐SCC‐104	**0.026**	NE	NE
SC263	**< 0.001**	NE	NE
SC263‐PBS	0.288	**< 0.001**	**< 0.001**
SC263‐R	**0.003**	NE	NE
SCC22b	0.997	**< 0.001**	**< 0.001**
SCC22b‐PBS	**0.008**	NE	NE
SCC22b‐R	**0.013**	NE	NE

Overall, treatment with afatinib under both normoxia and hypoxia established a significant G_0_/G_1_ cell cycle arrest. However, the influence of hypoxia on this treatment induced G_0_/G_1_ cell cycle arrest was cell line specific and independent of HPV status as well as cetuximab sensitivity.

#### 3.4.2 Treatment with afatinib leads to the induction of apoptotic cell death

**Figure 6:**
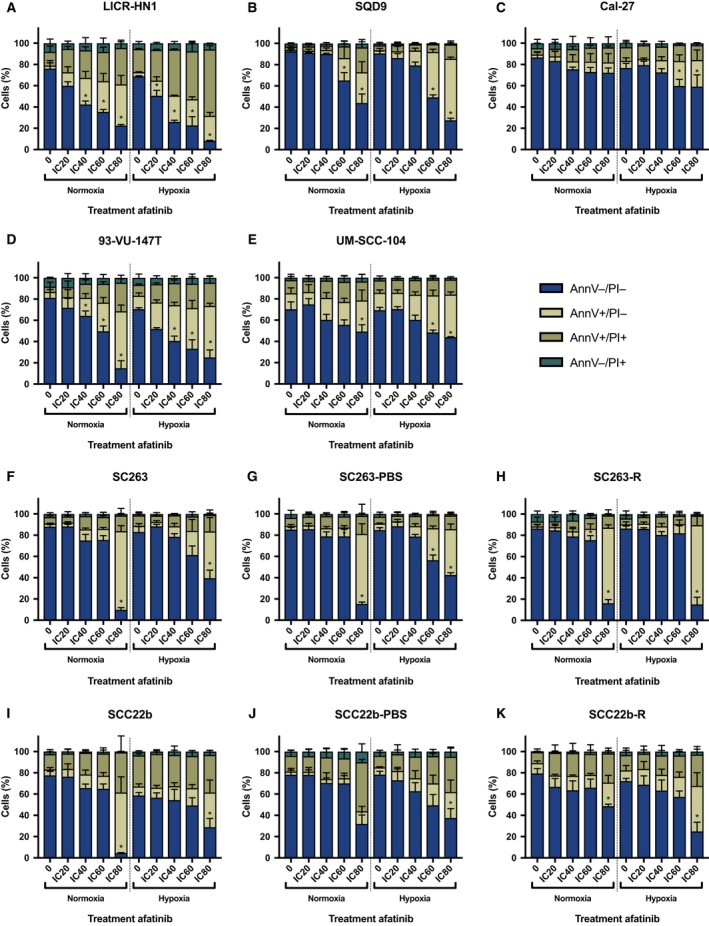
Induction of apoptotic cell death in HNSCC cell lines after afatinib treatment (0 nm, IC_20_, IC_40_, IC_60_, IC_80_) under both normoxic and hypoxic conditions. Cells were stained with annexin V‐FITC (AnnV) and PI and measured flow cytometrically. Treatment with afatinib induced an increase in the percentage of AnnV+/PI− and AnnV+/PI+ cells with a corresponding decrease of the percentage viable (AnnV−/PI−) cells in the majority of HPV‐negative (A–C) and HPV‐positive (D, E) intrinsically cetuximab‐resistant HNSCC cell lines under both normal and reduced oxygen conditions. This induction of apoptotic cell death was also observed in cetuximab‐sensitive (F, I) as well as acquired cetuximab‐resistant and PBS‐treated control (G, H, J, and K) HNSCC cell lines. *P‐value effect treatment on the percentage AnnV+/PI− cells ≤ 0.050.

**Figure 7:**
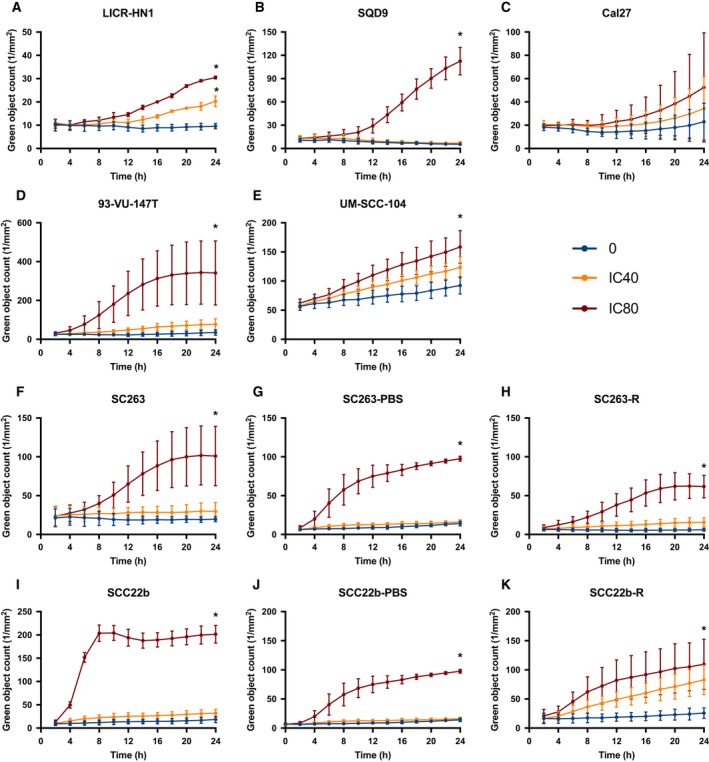
Caspase‐3/7 activity in HNSCC cell lines during afatinib treatment (0 nm, IC_40_, IC_80_) under normoxic conditions. The induction of apoptosis was detected by real‐time measurements of active caspase‐3/7 during afatinib treatment using the IncuCyte system. The green object count corresponds with the caspase‐3/7 activity. After 24 h, treatment with afatinib induced a significant increase in caspase‐3/7 activity in the majority of HPV‐negative (A–C) and HPV‐positive (D, E) intrinsically cetuximab‐resistant HNSCC cell lines. This significant increase in caspase‐3/7 activity was also detected in cetuximab‐sensitive (F, I), acquired cetuximab‐resistant, and PBS‐treated control (G, H, J, and K) HNSCC cell lines. *P‐value effect treatment on the green object count ≤ 0.050.

Besides the capacity of afatinib to induce a G_0_/G_1_ cell cycle arrest, we also assessed its ability to induce programmed apoptotic cell death using the AnnV/PI flow cytometric assay. This technique identifies cells in early (AnnV+/PI−) or late (AnnV+/PI+) phases of apoptosis. The majority of intrinsically cetuximab‐resistant HNSCC cell lines demonstrated a dose‐dependent increase in AnnV+/PI− and AnnV+/PI+ cells as well as a corresponding decrease in viable AnnV−/PI− cells after 72 h of treatment with afatinib under normal and reduced oxygen conditions (Fig. 6). However, in cetuximab‐sensitive, acquired cetuximab‐resistant, and PBS‐treated control HNSCC cell lines, afatinib induced an increase in AnnV+/PI− and AnnV+/PI+ cells after treatment with higher doses afatinib under both normoxia and hypoxia (Fig. 6). This induction of apoptotic cell death after treatment with afatinib under normoxia was confirmed by real‐time measurements of active caspase‐3/7 using the IncuCyte system. After 24 h of afatinib treatment, a significant dose‐depending increase in green object count, indicating an increased caspase‐3/7 activity, was observed in the majority of HNSCC cell lines (Fig. 7).

The effects of treatment and oxygen condition as well as their interaction on the percentage of AnnV−/PI−, AnnV+/PI−, and AnnV+/PI+ cells were determined using linear regression (Table 4). After 72 h of afatinib treatment, AnnV+ cells are mainly PI− and thus in the early phase of apoptosis. When looking at the viable cells and early apoptotic cells, the interaction between treatment and oxygen status was found significant in half of the HNSCC cell lines used in this study, indicating a cell line‐specific effect. Significant interaction indicated that the effect of afatinib treatment on the percentage of viable and early apoptotic is different under normoxia versus hypoxia. Moreover, it also implies that the effect of oxygen condition on the percentage of viable and early apoptotic cells varies after treatment with different concentrations afatinib. For example, the percentage of AnnV+/PI− cells was considerable high after treatment under hypoxia in comparison with treatment under normoxia in UM‐SCC‐104 cells. This is consistent with our previous findings in the cytotoxicity assay, indicating that the number of viable cells after afatinib treatment decreases more under hypoxic compared to normoxic conditions. When cells were already in late apoptosis after 72 h of treatment, no significant interaction between treatment and oxygen status was found in the majority of HNSCC cell lines used in this study (Table 4).

**Table 4:** *P*‐values for the interaction of treatment and oxygen condition on the percentage of AnnV−/PI−, AnnV+/PI−, and AnnV+/PI+ cells in HNSCC cell lines. If the interaction was not significant (*P* > 0.050), a model with the main effects of treatment and oxygen condition was fitted to test the significance of the effect of treatment and oxygen condition separately. NE: main effects were not estimated in case of a significant interaction between treatment and oxygen. Values ≤ ≤0.05 are indicated in bold

Cell line	*P*‐value interaction treatment and oxygen condition	*P*‐value main effect treatment	*P*‐value main effect oxygen condition
AnnV−/PI−			
LICR‐HN1	0.393	**< 0.001**	**< 0.001**
SQD9	**0.020**	NE	NE
Cal27	0.538	**< 0.001**	**< 0.001**
93‐VU‐147T	**0.001**	NE	NE
UM‐SCC‐104	0.694	**< 0.001**	0.053
SC263	**< 0.001**	NE	NE
SC263‐PBS	**< 0.001**	NE	NE
SC263‐R	0.682	**< 0.001**	0.342
SCC22b	**< 0.001**	NE	NE
SCC22b‐PBS	0.103	**< 0.001**	0.053
SCC22b‐R	**0.048**	NE	NE
AnnV+/PI−			
LICR‐HN1	**0.030**	NE	NE
SQD9	**0.002**	NE	NE
Cal27	0.296	**0.003**	**0.003**
93‐VU‐147T	**0.005**	NE	NE
UM‐SCC‐104	0.191	**< 0.001**	**0.003**
SC263	**< 0.001**	NE	NE
SC263‐PBS	**< 0.001**	NE	NE
SC263‐R	0.778	**< 0.001**	0.259
SCC22b	**0.005**	NE	NE
SCC22b‐PBS	0.371	**< 0.001**	**< 0.001**
SCC22b‐R	**0.042**	NE	NE
AnnV+/PI+			
LICR‐HN1	**0.007**	NE	NE
SQD9	**0.016**	NE	NE
Cal27	0.396	0.124	**0.023**
93‐VU‐147T	0.086	**< 0.001**	**0.023**
UM‐SCC‐104	0.682	0.090	0.209
SC263	0.531	**< 0.001**	0.339
SC263‐PBS	0.805	**0.017**	**0.286**
SC263‐R	0.870	0.092	0.538
SCC22b	0.130	**0.017**	**0.001**
SCC22b‐PBS	0.095	**< 0.001**	0.450
SCC22b‐R	0.653	**0.012**	0.769

### 3.5 Combining afatinib with cisplatin in HNSCC cell lines with different sensitivity to cetuximab shows additive to antagonistic effects

**Figure 8:**
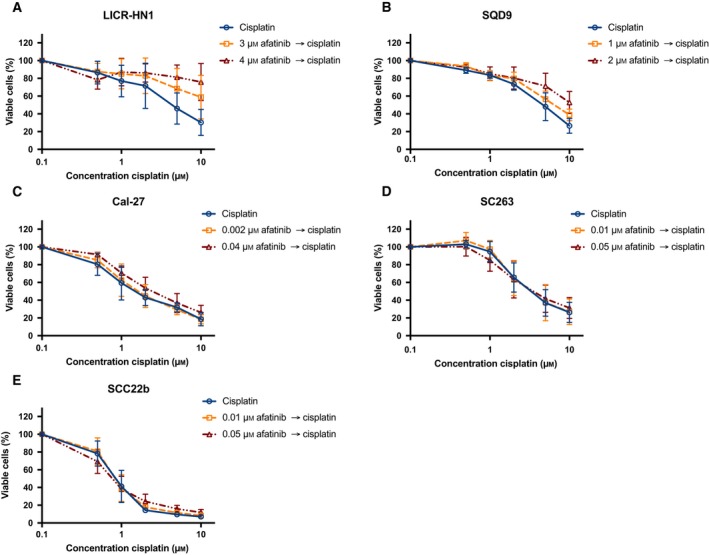
The cytotoxic effects of afatinib followed by cisplatin treatment in a panel of HNSCC cell lines with different sensitivity to cetuximab. Dose–response curves for the intrinsically cetuximab‐resistant cell lines LICR‐HN1 (A), SQD9 (B), and Cal‐27 (C) indicate an additive to antagonistic effect. Dose–response curves for the cetuximab‐sensitive cell lines SC263 (D) and SCC22b (E) show an additive to subadditive effect. Survival curves were corrected for the cytotoxic effect of 72‐h afatinib alone. Cells were treated with fixed concentrations afatinib, which were based on the outcome of the monotherapy experiments.

To investigate the potential interaction between afatinib and cisplatin, tumor cells were incubated with fixed doses of afatinib for 72 h combined with sequential treatment of 0–10 μm cisplatin for 24 h. The fixed afatinib concentrations were based on the outcome of the monotherapy experiments and correspond with the IC_20_ and IC_40_ values specific for each cell line. The dose–response curves of the cetuximab‐sensitive and intrinsically cetuximab‐resistant cell lines after treatment with these combination regimens are shown in Figs 7 and 8. All HNSCC cell lines were sensitive to treatment with cisplatin monotherapy with IC_50_ values ranging from 0.83 ± 0.11 μm to 4.65 ± 0.33 μm (Table 5). Compared to cisplatin treatment alone, treatment with afatinib before cisplatin demonstrated no significant decrease in IC_50_ value (*P* ≥ 0.149). In contrast, treatment with cisplatin followed by afatinib resulted generally in a significant increase in IC_50_ compared to the IC_50_ of cisplatin monotherapy (0.050 ≤ *P* ≤ 0.825). Furthermore, CI ranged from 1.01 ± 0.06 to 1.96 ± 0.40. Thus, sequential exposure to afatinib followed by cisplatin or the inverse regimen (i.e., cisplatin followed by afatinib) revealed additive or subadditive to antagonistic, yet no synergistic interactions.

**Figure 9:**
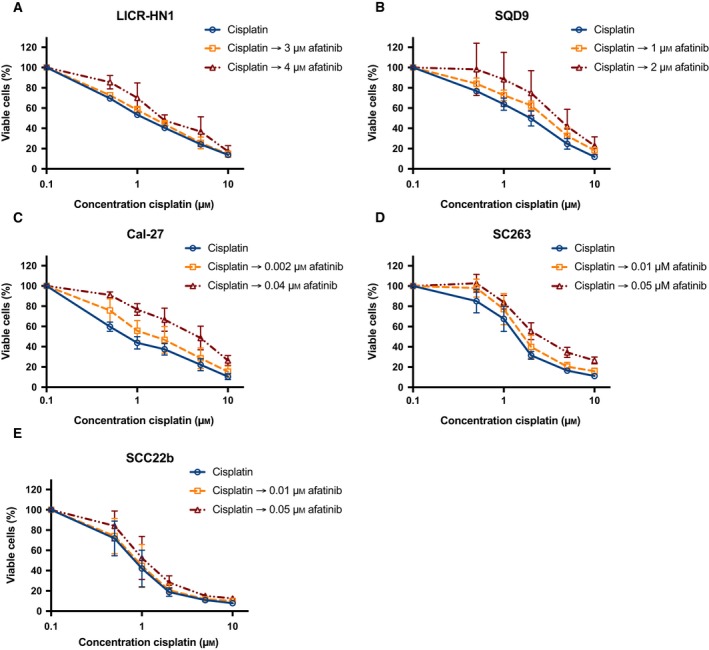
The cytotoxic effect of cisplatin followed by afatinib treatment in a panel of HNSCC cell lines with different sensitivity to cetuximab. Dose–response curves for the intrinsically cetuximab‐resistant cell lines LICR‐HN1 (A), SQD9 (B), and Cal‐27 (C) also indicate an additive to antagonistic effect. Dose–response curves for the cetuximab‐sensitive cell lines SC263 (D) and SCC22b (E) show an additive to antagonistic effect. Survival curves were corrected for the cytotoxic effect of 72‐h afatinib alone. Cells were treated with fixed concentrations afatinib, which were based on the outcome of the monotherapy experiments.

**Table 5:** IC_50_, CI, and standard errors for HNSCC cell lines after sequential treatment with afatinib followed by cisplatin as well as cisplatin followed by afatinib. CI < 0.8, CI = 1.0 ± 0.2, and CI > 1.2 indicated synergism, additivity, or antagonism, respectively. ND, not determined, as cell survival did not decrease below 50%. *P* ≤ 0.050, significant difference in IC_50_ compared to cisplatin monotherapy. *P*‐values ≤ ≤0.05 are indicated in bold. –, cannot be calculated

Cell line	Condition	IC_50_ (μm)	*P*‐value	CI
LICR‐HN1	72 h 0 μm afatinib → 24 h cisplatin	4.42 ± 0.49	–	–
	72 h 3 μm afatinib → 24 h cisplatin	ND	–	1.34 ± 0.38
	72 h 4 μm afatinib → 24 h cisplatin	ND	–	1.50 ± 0.65
	24 h cisplatin → 72 h 0 μm afatinib	1.25 ± 0.05	–	–
	24 h cisplatin → 72 h 3 μm afatinib	1.48 ± 0.04	**0.050**	1.07 ± 0.02
	24 h cisplatin → 72 h 4 μm afatinib	2.25 ± 0.37	**0.050**	1.30 ± 0.13
SQD9	72 h 0 μm afatinib → 24 h cisplatin	4.65 ± 0.33	–	–
	72 h 1 μm afatinib → 24 h cisplatin	6.58 ± 0.61	0.149	1.16 ± 0.185
	72 h 2 μm afatinib → 24 h cisplatin	ND	–	1.33 ± 0.42
	24 h cisplatin → 72 h 0 μm afatinib	1.78 ± 0.14	–	–
	24 h cisplatin → 72 h 1 μm afatinib	2.78 ± 0.28	**0.050**	1.27 ± 0.17
	24 h cisplatin → 72 h 2 μm afatinib	4.06 ± 0.16	**0.050**	1.56 ± 0.25
Cal‐27	72 h 0 μm afatinib → 24 h cisplatin	1.72 ± 0.28	–	–
	72 h 0.002 μm afatinib → 24 h cisplatin	1.83 ± 0.28	0.465	1.01 ± 0.06
	72 h 0.04 μm afatinib → 24 h cisplatin	2.68 ± 0.49	0.175	1.23 ± 0.11
	24 h cisplatin → 72 h 0 μm afatinib	0.83 ± 0.11	–	–
	24 h cisplatin → 72 h 0.002 μm afatinib	1.58 ± 0.19	0.127	1.31 ± 0.09
	24 h cisplatin → 72 h 0.04 μm afatinib	4.07 ± 0.48	**0.050**	1.96 ± 0.40
SC263	72 h 0 μm afatinib → 24 h cisplatin	3.48 ± 0.64	–	–
	72 h 0.01 μm afatinib → 24 h cisplatin	3.44 ± 0.78	1.000	1.02 ± 0.02
	72 h 0.05 μm afatinib → 24 h cisplatin	3.72 ± 0.71	0.827	1.03 ± 0.12
	24 h cisplatin → 72 h 0 μm afatinib	1.44 ± 0.20	–	–
	24 h cisplatin → 72 h 0.01 μm afatinib	1.82 ± 0.35	0.513	1.24 ± 0.11
	24 h cisplatin → 72 h 0.05 μm afatinib	2.87 ± 0.71	**0.050**	1.73 ± 0.51
SCC22b	72 h 0 μm afatinib → 24 h cisplatin	0.87 ± 0.09	–	–
	72 h 0.01 μm afatinib → 24 h cisplatin	0.88 ± 0.13	0.602	1.13 ± 0.13
	72 h 0.05 μm afatinib → 24 h cisplatin	0.80 ± 0.17		1.37 ± 0.42
	24 h cisplatin → 72 h 0 μm afatinib	0.84 ± 0.09	–	–
	24 h cisplatin → 72 h 0.01 μm afatinib	0.90 ± 0.12	0.825	1.11 ± 0.10
	24 h cisplatin → 72 h 0.05 μm afatinib	1.15 ± 0.20	0.268	1.38 ± 0.18

## 4 Discussion

Therapeutic resistance to EGFR‐targeted therapies remains a major clinical problem. In order to overcome resistance to these EGFR‐targeted therapies, new treatment options are urgently needed. Due to extensive crosstalk among HER receptors, blockade of one HER receptor can be partially compensated by other HER family members, which therefore must be targeted by new therapeutic regimens. In contrast to the first‐generation EGFR inhibitors, afatinib blocks irreversibly EGFR, HER2, and HER4. As a result, we hypothesized that treatment with afatinib might result in a distinct and more pronounced therapeutic benefit. To test this hypothesis, we investigated the cytotoxicity of afatinib in a panel of two HPV‐positive and nine HPV‐negative HNSCC cell lines that are either sensitive or intrinsically/acquired resistant to cetuximab.

We observed that the majority of HNSCC cell lines used in this study demonstrated high expression of EGFR, HER2, and HER3, but rather low HER4 expression under both normal and reduced oxygen conditions. It has already been established that EGFR is a key survival factor under hypoxic conditions, as EGFR stimulates HIF signaling to improve cellular survival (Wouters *et al*., 2013). On the other hand, HIF signaling can also activate the EGFR pathway, potentiating survival and tumor growth (Wang and Schneider, 2010). Consistent with previous studies, we demonstrated that the percentage of EGFR‐positive cells and the EGFR expression level was significantly increased under reduced oxygen conditions (Krause *et al*., 2005; Laderoute *et al*., 1992; Swinson and O'Byrne, 2006). Besides the effect on EGFR expression, hypoxia also induced a significant raise in HER2 expression. In contrast, a significant decrease in the percentage of HER3‐positive cells but not in HER3 expression level was observed under hypoxia. No effect of hypoxia on HER4 expression was noticed.

Previous research demonstrated that EGFR is expressed in more than 90% of HNSCC and that high EGFR expression is correlated with worse outcomes (Ang *et al*., 2002; Cohen, 2006; Dassonville *et al*., 1993; Rubin Grandis *et al*., 1996, 1998; Santini *et al*., 1991). However, EGFR expression level and gene copy number are not predictive for response to treatment with EGFR‐targeted therapies plus platinum/5‐fluorouracil as first‐line therapy for patients with R/M HNSCC (Licitra *et al*., 2011, 2013). Furthermore, increased HER2 and HER3 expression have been associated with gefitinib resistance (Erjala *et al*., 2006). Thus, cetuximab resistance may arise from alterations in the expression level of HER receptors. In our study, no significant changes in expression of HER receptors were found between cetuximab‐sensitive, intrinsically cetuximab‐resistant, and acquired cetuximab‐resistant HNSCC cell lines. Nevertheless, the kinase activity of these receptors can still be strongly induced in cetuximab‐resistant cells (Wheeler *et al*., 2008), making inhibition of homo‐ and heterodimerization of these HER receptors still a promising strategy to overcome cetuximab resistance.

Overall, as the majority of our HNSCC cell lines demonstrated high EGFR, HER2, and HER3 expression under normal and reduced oxygen conditions, these intrinsically cetuximab‐resistant and acquired cetuximab‐resistant HNSCC cell lines are valid target candidates for afatinib treatment.

In the current study, we demonstrated that afatinib indeed has the potential to overcome intrinsic and acquired cetuximab resistance in both HPV‐positive and HPV‐negative HNSCC tumors, as it was able to establish a cytotoxic effect in HNSCC cell lines with different cetuximab sensitivity and HPV status. No significant effect of cetuximab resistance status and HPV status on the cytotoxic effect of afatinib was observed. Our results are in concordance with those of others who showed that targeting multiple members of the HER receptor family is effective in overcoming intrinsic and acquired cetuximab resistance (Iida *et al*., 2014, 2016; Quesnelle and Grandis, 2011). Furthermore, a phase II study has recently demonstrated that afatinib showed antitumor activity comparable to cetuximab with lack of cross‐resistance (Seiwert *et al*., 2014). Thus, inhibition of multiple members of the HER receptor family seems to be necessary in order to completely overcome intrinsic and acquired cetuximab resistance. Nevertheless, previous research by us and others demonstrated that MEHD7945A, a dual mAb targeting EGFR and HER3, is only partially able to overcome cetuximab resistance in HNSCC cell lines (De Pauw *et al*., 2017; Fayette *et al*., 2016). These studies suggested the presence of additional resistance mechanisms to cetuximab treatment besides HER3 signaling. As afatinib irreversibly inhibits EGFR, HER2 as well as HER4 and consequently also HER3 by inhibiting its dimerization partners, a more pronounced therapeutic benefit with afatinib might be expected in patients with HNSCC experiencing cetuximab resistance.

Despite that statistical analysis did not reveal a significant influence of cetuximab resistance on the cytotoxicity of afatinib, our results indicated the possibility of cross‐resistance between cetuximab and afatinib. The presence of cross‐resistance between cetuximab and afatinib indicates that resistance to EGFR inhibitors is not exclusively due to alterations of HER receptor signaling.

In the LUX‐Head and Neck 1 phase III trial, progression‐free survival was significantly improved by afatinib compared to methotrexate in patients with second‐line R/M HNSCC (Machiels *et al*., 2015). In contrast to the study of Seiwert *et al*. (2014), subgroup analysis showed a pronounced therapeutic benefit for patients who had not been treated with an EGFR‐targeted antibody in R/M setting, indicating cross‐resistance with afatinib. The lack of an observed overall survival benefit with afatinib compared to methotrexate might result from some characteristics of the study population, particularly the inclusion of HPV‐positive patients and those who received previous EGFR‐targeted treatment. The inclusion of these unselected patients might have diluted the treatment effect of afatinib (Machiels *et al*., 2015). Indeed, further subgroup analysis of the LUX‐Head and Neck 1 trial identified biomarkers (HPV‐negative, EGFR‐amplified, low HER3 expression, and high PTEN expression) that could predict clinical outcomes with afatinib versus methotrexate in R/M HNSCC. This finding emphasizes the importance of well‐defined biomarkers for optimal patient selection.

Interestingly, afatinib demonstrated higher cytotoxic activity under hypoxia in cetuximab‐sensitive, intrinsically cetuximab‐resistant, and acquired cetuximab‐resistant HNSCC cell lines. This is an important finding, as oxygen deficiency is a common characteristic of HNSCC and these hypoxic tumor regions often contain viable cells that are more resistant to conventional chemotherapy and/or radiotherapy (Wouters *et al*., 2007). It has already been speculated that hypoxia enhances the sensitivity to the cytotoxic effect of EGFR‐targeted mAb and TKIs, given the link between HIF and EGFR signaling (Boeckx *et al*., 2015b; Pore *et al*., 2006; Riesterer *et al*., 2011). For instance, cetuximab and gefitinib are able to overcome hypoxia‐induced drug resistance by downregulation of HIF‐1alpha through inhibition of the EGFR–Akt pathway (Li and Fan, 2010; Li *et al*., 2008b; Luwor *et al*., 2005; Rho *et al*., 2009). Hence, treatment with afatinib can similarly lead to HIF‐1alpha downregulation. Increased expression of EGFR and/or HER2 under hypoxic conditions may also explain afatinib's increased cytotoxic effect under hypoxia, as afatinib irreversibly inhibits both receptors.

Concerning the mechanism of action by with afatinib exerts its effect, we observed that treatment with afatinib under both normal and reduced oxygen levels established a G_0_/G_1_ cell cycle arrest and induction of apoptotic cell death in the majority of cetuximab‐sensitive, intrinsically cetuximab‐resistant, and acquired cetuximab‐resistant HNSCC cell lines, which is in accordance with previous findings in HNSCC (Iida *et al*., 2016; Liu *et al*., 2016; Macha *et al*., 2017). However, induction of G_0_/G_1_ cell cycle arrest and induction of apoptotic cell death were not associated with each other in all cell lines under investigation. As activation of the EGFR signal transduction pathway can result in stimulation of both proliferation and anti‐apoptotic signaling (Wee and Wang, 2017), blockade of EGFR as well as other HER family members through afatinib can prevent subsequent activation of these signaling pathway. Consequently, afatinib can affect the cell cycle, programmed cell death, or both cellular processes simultaneously.

Importantly, in the current study, we also included HPV status of our HNSCC cell line panel as an important variable. Despite the impressive progress regarding comprehension of the etiology, epidemiology, and prognostic impact of HPV, the extent to which HPV status may be predictive of response to therapeutic regimens used in the treatment of HNSCC remains incompletely understood (Vermorken, 2017).

Previous research demonstrated that the expression of EGFR was significantly increased in HPV‐negative HNSCC tumors, whereas the expression of HER2 and HER3 was significantly elevated in HPV‐positive HNSCC, suggesting that agents targeting multiple HER receptors might be more effective in HPV‐positive HNSCC (Mazibrada *et al*., 2014; Pollock *et al*., 2015). These findings were supported by data available from the Cancer Genome Atlas Network and Chicago Genomics Cohort, demonstrating that HPV‐negative tumors show high EGFR levels and EGFR amplification, whereas HPV‐positive tumors show generally low EGFR expression and high HER2 and HER3 expression (Pollock *et al*., 2015; Szturz *et al*., 2017). In contrast, we did not find significant differences in EGFR, HER2, and HER3 expression between the HPV‐positive and HPV‐negative HNSCC cell lines included in our extensive panel of HNSCC cell lines. Hence, HPV did not induce overexpression of HER2 and HER3 in our HPV‐positive HNSCC cell lines. Yet, it is important to keep in mind that the use of only two HPV‐positive cell lines limits our interpretations. Nevertheless, in our as well as Pollock's study (Pollock *et al*., 2015), afatinib established a clear cytotoxic effect after drug exposure in HPV‐positive HNSCC cell lines, which might indicate its potential for the treatment of HPV‐positive patients with HNSCC.

A phase Ib trial recently demonstrated that afatinib, ribavirin, and weekly paclitaxel and carboplatin as induction chemotherapy is safe and well tolerated in patients with locally advanced HPV‐associated oropharyngeal HNSCC (Dunn *et al*., 2017). In contrast, clinical data of the LUX‐Head and Neck 1 trial do not support these preclinical observations and demonstrated that patients with HPV‐positive tumors had less benefit from treatment with afatinib (Machiels *et al*., 2015, 2016). They reported that subgroups of patients with HNSCC, who may achieve increased benefit from afatinib, were identified based on prespecified tumor biomarkers (i.e., HPV‐negative, EGFR amplification, low HER3 expression, and high PTEN expression (Cohen *et al*., 2017). Consequently, further preclinical and clinical research is needed to draw final conclusions upon the possible predictive role of HPV status for the treatment with EGFR‐targeted therapies.

At the moment, most cancer treatments are combinations of chemotherapeutic agents and/or radiotherapy, and it is expected that new EGFR‐targeted agents will achieve their greatest efficacy in combination with traditional cytotoxic agents and/or radiotherapy. Indeed, previous research demonstrated that afatinib radiosensitizes HNSCC cells by targeting cancer stem cells (Macha *et al*., 2017). Furthermore, concurrent treatment of afatinib with gemcitabine demonstrated synergistic antitumor effects in nasopharyngeal carcinoma, but also potentially enhanced toxicity in mouse models (Xue *et al*., 2016). As cetuximab has been approved for the treatment of R/M HNSCC in combination with platinum‐based drugs (Vermorken *et al*., 2008), we investigated the combination of afatinib with cisplatin. Preclinical research in wild‐type EGFR HNSCC cell lines already demonstrated that cotreatment with afatinib enhances the therapeutic effect of platinum‐based chemotherapy such as cisplatin (Brands *et al*., 2016). However, it was also reported that simultaneous treatments with afatinib and standard chemotherapy are associated with increased frequency of side effects in HNSCC xenograft studies and clinical trials, so that we found it more relevant to study sequential treatment regimens (Chung *et al*., 2016; Xue *et al*., 2016). Unfortunately, in our study, we were not able to demonstrate any synergistic interaction upon sequential treatment of afatinib followed by cisplatin or the inverse sequence, being cisplatin followed by afatinib. In contrast to cetuximab, afatinib seems not the ideal combination partner with platinum‐based drugs for the treatment of HNSCC.

Besides combining afatinib with standard chemotherapy and/or radiotherapy, combinations with other targeted agents such as CDK and Akt inhibitors have shown synergistic effects in HNSCC cell lines (Beck *et al*., 2016; Silva‐Oliveira *et al*., 2017). However, combining two EGFR inhibitors, that is, afatinib with cetuximab, did not reveal any advantage to single‐agent treatment (Quesnelle and Grandis, 2011; Young *et al*., 2015). Although a randomized phase II of afatinib versus cetuximab in R/M HNSCC suggested a lack of cross‐resistance between afatinib and cetuximab (Seiwert *et al*., 2014), biomarker analysis of the LUX‐Head and Neck 1 trial suggested that afatinib is more effective in patients whose tumors are cetuximab naïve, indicating the possibility of cross‐resistance (Cohen *et al*., 2017).

Of particular interest and complexity are regimens combining immunotherapy with EGFR‐targeted therapy in HNSCC. The EXTREME study demonstrated that cetuximab could prolong median overall survival when added to platinum/5‐fluorouracil doublet in R/M HNSCC (Vermorken *et al*., 2008). To date, however, no other EGFR‐blocking agent has demonstrated these results in clinical studies (Szturz and Vermorken, 2017). From this point of view, it appears that cetuximab has additional immune‐based mechanisms of activity through stimulation of antibody‐dependent cytotoxicity and enhancement of cytotoxic T‐lymphocyte cross‐priming by dendritic cells (Kimura *et al*., 2007; Yang *et al*., 2013). The PD‐1‐directed immune checkpoint inhibitors, nivolumab (based on phase III data) and pembrolizumab (based on phase II data), are novel therapeutic agents that have gained FDA approval and have become available for second‐line treatment of R/M HNSCC (Chow *et al*., 2016; Ferris *et al*., 2016; Seiwert *et al*., 2016). There are several clinical studies exploring the inhibition of the PD‐1/PD‐L1 axis in combination with or without cetuximab (NCT02764593 and NCT02999087).

As afatinib has no additional immune‐based mechanisms, it seems not the ideal combination partner with immunotherapy. Nevertheless, afatinib is able to overcome cetuximab resistance, as shown in this study. Several clinical studies are currently evaluating single‐agent treatment with afatinib in HNSCC (LUX‐Head and Neck 2, 3, and 4, that is, NCT0134566, NCT01856478, and NCT02131155). Furthermore, afatinib displayed a radiosensitizing effect in preclinical studies (Huguet *et al*., 2016; Macha *et al*., 2017). As a result, afatinib is still considered as a promising agent to treat patients with HNSCC. However, optimization of combination treatment regimens with afatinib and conventional as well as other targeted therapies is necessary. Furthermore, identifying predictive biomarkers to select the patients that benefit most from these particular combination strategies is of crucial importance.

## 5 Conclusion

Our results suggest that afatinib has the potential to overcome intrinsic and acquired cetuximab resistance in both HPV‐positive and HPV‐negative HNSCC tumors, as it was able to establish a cytotoxic effect in HNSCC cell lines with different cetuximab sensitivity and HPV status. However, cross‐resistance between cetuximab and afatinib might be possible. Therefore, further research is required to identify predictive biomarkers to optimize patient selection. Treatment with afatinib causes a G_0_/G_1_ cell cycle arrest and induces apoptotic cell death. Sequential combinations of afatinib with cisplatin demonstrated additive to antagonistic effects. In contrast to cetuximab, afatinib seems not to be the ideal combination partner with platinum‐based drugs for the treatment of HNSCC. In this study, neither cetuximab resistance nor HPV status significantly influenced the expression of HER family members in HNSCC cell lines. In contrast, the expression of EGFR, HER2, and HER3 was significantly altered under reduced oxygen conditions. Furthermore, the cytotoxic effect of afatinib was increased under hypoxia. Overall, these data support the hypothesis that afatinib might be a promising therapeutic strategy to treat patients with HNSCC experiencing intrinsic or acquired cetuximab resistance. Nevertheless, further identification of predictive biomarkers is necessary.

## Author contributions

IDP participated in the design of the study, carried out the *in vitro* experiments, performed statistical analysis, and drafted the manuscript. FL obtained funding for the study, participated in the design, and helped to draft the manuscript. JVB assisted in the *in vitro* experiments, participated in its design, and contributed to draft the manuscript. HB assisted in the *in vitro* experiments toward his Master thesis in Biomedical Sciences. EF performed statistical analysis. VD and PP participated in the design of the study. MP obtained funding for the study. JBV participated in the design of the study and helped to draft the manuscript. AW conceived of the study, participated in its design and coordination, and assisted to draft the manuscript. All authors read and approved the final manuscript.

## Conflict of interest

JBV participated in advisory boards of Boehringer‐Ingelheim. The other authors declare that they have no conflict of interest.
